# Familial risk and childhood adversity interplay in the onset of psychosis

**DOI:** 10.1192/bjpo.bp.115.000158

**Published:** 2015-06-23

**Authors:** Antonella Trotta, Marta Di Forti, Conrad Iyegbe, Priscilla Green, Paola Dazzan, Valeria Mondelli, Craig Morgan, Robin M. Murray, Helen L. Fisher

**Affiliations:** **Antonella Trotta**, PhD, **Marta Di Forti**, PhD, **Conrad Iyegbe**, PhD, **Priscilla Green**, PhD, **Paola Dazzan**, MRCPsych, Department of Psychosis Studies, Institute of Psychiatry, Psychology & Neuroscience, King's College London, UK; **Valeria Mondelli**, PhD, Section of Stress, Psychiatry and Immunology and Perinatal Psychiatry, Department of Psychological Medicine, Institute of Psychiatry, Psychology & Neuroscience, King's College London, UK; **Craig Morgan**, PhD, Health Service and Population Research Department, Institute of Psychiatry, Psychology & Neuroscience, King's College London, UK; **Robin M. Murray**, FRCPsych, Department of Psychosis Studies, Institute of Psychiatry, Psychology & Neuroscience, King's College London, UK; **Helen L. Fisher**, PhD, MRC Social, Genetic and Developmental Psychiatry Centre, Institute of Psychiatry, Psychology & Neuroscience, King's College London, UK

## Abstract

**Background:**

The association between childhood adversity and psychosis in adulthood is well established. However, genetic factors might confound or moderate this association.

**Aims:**

Using a catchment-based case–control sample, we explored the main effects of, and interplay between, childhood adversity and family psychiatric history on the onset of psychosis.

**Method:**

Childhood adversity (parental separation and death, physical and sexual abuse) was assessed retrospectively in 224 individuals with a first presentation of psychosis and 256 community controls from South London, UK. Occurrence of psychotic and affective disorders in first-degree relatives was ascertained with the Family Interview for Genetic Studies (FIGS).

**Results:**

Parental history of psychosis did not confound the association between childhood adversity and psychotic disorder. There was no evidence that childhood adversity and familial liability combined synergistically to increase odds of psychosis beyond the effect of each individually.

**Conclusions:**

Our results do not support the hypothesis that family psychiatric history amplifies the effect of childhood adversity on odds of psychosis.

**Declaration of interest:**

None.

**Copyright and usage:**

© The Royal College of Psychiatrists 2015. This is an open access article distributed under the terms of the Creative Commons Attribution (CC BY) licence.

Childhood adversity has been shown to be associated with early psychotic symptoms,^1–3^ with transition to clinical psychosis in individuals at ultra-high risk (UHR),^4^ as well as with the onset of a full-blown psychotic disorder.^5–7^ Furthermore, associations are usually stronger with increasing frequency and severity of the trauma experienced,^8^ indicating a key role for this environmental factor in the development of psychosis. However, genetic vulnerabilities have been repeatedly shown to be involved in the aetiology of psychosis.^9^ Studies have estimated the heritability of schizophrenia to be around 60%,^10^ and concordance for schizophrenia in monozygotic twins is around 50%.^11^ Moreover, having one or more biological parents with a history of psychosis has been associated with a greater risk of exposure to stressful life events and adverse experiences during childhood^12,13^ and also with the development of psychotic symptoms and disorders.^14,15^ This suggests that a ‘passive’ type of gene–environment correlation (rGE)^16^ might be operating such that parents provide their children with both an adverse upbringing and a genetic vulnerability to developing psychosis. This implies that parents’ genetic make-up may be confounding the childhood adversity–psychosis associations observed in previous studies.

It is also possible that genetic factors moderate the association between childhood adversity and psychosis (a gene–environment interaction, G × E),^16^ potentially by influencing how an individual reacts biologically and/or psychologically following exposure to adversity which may set them off on the path to psychosis.^17^ A number of studies have examined rGE and G × E using indirect measures of genetic risk, such as being a relative, a twin or adopted-away offspring of a person with schizophrenia.^18–20^ The advantage of using familial liability to psychosis as a proxy for genetic risk is that it may capture a greater proportion of genetic load, including gene–gene interactions, in contrast to studies using direct molecular genetic measures that tend to incorporate only a small contribution to genetic variation in the form of single-nucleotide polymorphisms (SNPs).^21^ Moreover, despite the advent of polygenic risk scores, which combine multiple SNPs and thus increase the amount of genetic variation accounted for, these may not provide any additional mechanistic clues over and above measures of family psychiatric history because they aggregate information across thousands of SNPs, thus making it difficult to disentangle which combinations of SNPs are driving the interaction.^22^ It is important to note though that a history of psychosis and other psychiatric disorders in first-degree relatives is only a proxy for genetic risk and may also reflect some aspects of the environment in which individuals are brought up,^17^ though this component is likely to be fairly small.^10^ Therefore, at present there is no ideal measure of genetic risk to employ in exploring rGE and G × E for psychosis and thus triangulation of evidence obtained from different measures across multiple studies is likely to be the best overall strategy. Here we focus on trying to broaden the evidence base in relation to familial liability to psychosis.

Despite several studies previously investigating interactions between genetic liability and childhood adversity in the onset of psychosis, those involving familial liability as a measure of proxy genetic risk have been restricted to general population samples.^1,14,23–26^ Only one study has investigated the interplay between childhood adversity and familial risk for mental health problems in a first-episode psychosis sample,^27^ but this focused solely on one form of adversity, namely maternal physical abuse. In light of this, the aim of the present study was to extend existing research by investigating, for the first time, the interplay between various forms of childhood adversity and family psychiatric history in the onset of psychotic disorders. We sought to test two hypotheses: (a) individuals with a parental history of psychosis or affective disorders would have a greater prevalence of both psychotic disorders and childhood adversity than those without this proxy genetic vulnerability; and (b) childhood adversity would combine synergistically (on an additive scale) with familial liability to increase the odds of psychotic disorder.

## Method

### Participants

The sample was drawn from patients who participated in the Genes and Psychosis (GAP) study from Lambeth, Southwark and Croydon adult in-patient units of the South London & Maudsley (SLAM) Mental Health National Health Service (NHS) Foundation Trust, UK. Inclusion criteria for patients were: age 16–65 years, presenting to psychiatric services for the first time with a psychotic disorder (codes F20-29 and F30-33 from the International Classification of Diseases (ICD-10)),^28^ and resident within tightly defined catchment areas in south-east London, UK. Exclusion criteria were: organic psychosis; IQ < 50; previous contact with services for psychosis; and transient psychotic symptoms resulting from acute intoxication (ICD-10).^28^ ICD-10 diagnoses were determined using data from the Schedules for Clinical Assessment in Neuropsychiatry (SCAN).^29^

Control participants were aged 16–65 years and recruited from the local population living in the area served by the Trust, by means of internet and newspaper advertisements, and distribution of leaflets at train stations, shops and job centres. Efforts were made to obtain a control sample that was representative of the general population in age, gender, ethnicity, educational qualifications and employment status. The Psychosis Screening Questionnaire (PSQ)^30^ was administered to all potential control group participants; individuals were excluded if they met criteria for a psychotic disorder.

### Measures

A range of sociodemographic information was obtained including age at interview, gender, current level of education and self-ascribed ethnicity (using the UK 2001 census categories) during face-to-face interviews using the Medical Research Council Sociodemographic Schedule.^31^

### Childhood adversity

The Childhood Experience of Care and Abuse Questionnaire (CECA-Q)^32^ was employed to retrospectively elicit information from participants concerning a range of adverse childhood experiences before the age of 17 years. For this analysis, physical abuse by the main mother and father figures (usually but not necessarily the biological parents), sexual abuse by any adult or an individual at least 5 years older than the recipient, separation from a parent for at least 6 months and death of a parent were included. Full details of the questionnaire are provided in Bifulco *et al*.^32^ The CECA-Q has been shown to have good internal consistency,^33^ satisfactory levels of test–retest reliability over 7 years in a psychosis sample^34^ and reasonable concurrent validity with existing measures.^32–34^ The CECA-Q elicits concrete examples of adverse experiences, and severity of the responses is scored in a standardised manner to enhance validity of the self-reported experiences.^32^ Every childhood experience section of the CECA-Q begins with screening questions and then positive responses are followed up with more detailed questions. This questionnaire was read out to all participants to improve the accuracy of the fixed category responses obtained. The physical and sexual abuse variables were dichotomised into severe and non-severe categories using the most conservative published cut-off points.^32^

### Family history of mental illness

The Family Interview for Genetic Studies (FIGS; https://www.nimhgenetics.org/interviews/figs) was used to obtain information about the participant’s family history of mental health problems. This interview begins with a brief construction of a pedigree diagram for the participant’s first-degree relatives and a series of screening questions to elicit information about possible mental health problems in these relatives. Positive responses to any of these are followed up with more specific questions to obtain symptom and treatment information for each potentially affected relative. Only three of these supplementary sections were chosen for this study, namely depression, mania and psychosis. For patients, this interview was supplemented by information retrieved from clinical records. To maximise genetic risk, only information on first-degree relatives (participant’s biological mother and father, full siblings and children) was used. The FIGS consensus diagnoses were divided into several familial risk variables. First, a ‘family mental illness’ variable referred to the presence (1) or absence (0) of current or past psychosis, mania or depression in at least one first-degree relative. A ‘family psychosis’ variable denoted the presence (1) or absence (0) of a current or previous diagnosis of psychosis in at least one first-degree relative. A ‘parental mental illness’ variable was also created that indicated the presence (1) or absence (0) of a current or previous diagnosis of psychosis, mania or depression in at least one biological parent. Similarly, a variable for ‘parental psychosis’ was created that denoted the presence (1) or absence (0) of current or past psychosis in at least one biological parent.

### Ethics

Ethical permission was obtained from the SLAM and the Institute of Psychiatry Research Ethics Committee. All participants provided written consent after reading a detailed information sheet.

### Statistical analysis

All analyses were performed using Stata version 11.1 (Stata-Corp, College Station, TX, USA). First, main effects of each type of childhood adversity and (general and psychotic) family mental illness on psychosis caseness were assessed using a series of logistic regressions. Second, we tested whether differences in an individual’s proxy genetic liability might drive differential environmental exposure. Specifically, the passive type of rGE was explored using binary logistic regression analysis to estimate odds ratios (ORs) of the associations between history of parental mental illness or parental psychosis and (a) psychotic disorder in the participants, and (b) each subtype of childhood adversity. If familial liability is associated with both disorder and adversity, then this indicates the possibility of a passive rGE (albeit a ‘proxy gene’ by environment correlation). The possibility that parental psychopathology may attenuate the association between childhood adversity and psychosis was also addressed by rerunning the association between childhood adversity and psychotic disorder with parental history of psychosis added as a confounder.

Next, we examined whether there was evidence that childhood adversity combined synergistically with each type of familial liability by testing for interaction on an additive scale using interaction contrast ratios (ICRs).^35,36^ This approach uses ORs to estimate the relative excess risk due to interaction. Biological synergism (the odds of psychosis among individuals with both risk factors being greater than the sum of the independent effects of each risk factor) can be better estimated from additive statistical interaction than multiplicative statistical interaction.^37,38^ As the numbers of cases and controls with a family history of psychosis were very small, interaction analyses were only conducted for family and parental history of mental illness. *Post hoc* estimations of power and sample size were estimated using the ‘sampsi’ command in Stata 11. All analyses were controlled for gender (male or female), age at interview (16–64 years), ethnicity (White British, Black Caribbean, Black African, Asian [all], Mixed or Other) and level of education (no qualification, school-leaving qualifications, A-levels/vocational/college or university/professional qualifications).

## Results

Information on family history of mental illness was available on 224 of the 285 patients and 250 of the 256 controls with a completed CECA-Q. The patients with and without FIGS data did not differ in terms of gender (χ^2^ = 0.003, *P* = 1.000), age (*t* = 0.587, *P* = 0.558) or diagnosis (χ^2^ = 0.184, *P* = 1.000). The basic demographic data by case and control status for those included in the analyses are presented in Table 1.

**Table 1:** Basic demographic characteristics of psychosis patients and unaffected controls

Demographic variable	Patients (*n* = 285) *n* (%)	Controls (*n* = 256) *n* (%)	χ^2^	d.f.	*P*
Gender			2.57	1	0.065
Male	172 (60.4)	137 (53.5)			
Female	113 (39.6)	119 (46.5)			

Ethnicity			32.60	5	<0.001
White British	72 (25.3)	102 (39.9)			
Black Caribbean	56 (19.6)	39 (15.2)			
Black African	65 (22.9)	32 (12.5)			
White other	30 (10.5)	50 (19.5)			
Asian (all)	24 (8.4)	16 (6.3)			
Other	38 (13.3)	17 (6.6)			

Level of education			76.73	4	<0.001
No qualifications	48 (17.6)	7 (3.0)			
School leaving qualifications	64 (23.5)	23 (10.0)			
A-levels/college level qualifications	40 (14.7)	53 (22.9)			
Vocational qualifications	66 (24.3)	37 (16.0)			
University or professional qualifications	54 (19.9)	111 (48.1)			

Age in years			*t*=0.342	536	0.733
Mean (s.d.)	28.9 (9.3)	29.2 (9.9)			

d.f., degrees of freedom; s.d., standard deviation.

More than half of the patients were male (60.4%) and from Black or other minority ethnic groups (BME; 74.7%). The majority of the controls were also male (53.5%) and from BME groups (60.1%). Mean age at interview was around 29 years both for patients and controls. As expected, patients were significantly more likely to be from a BME group (*P* < 0.001), and have none or only school-leaving qualifications (*P* < 0.001) compared with controls. There was no significant difference in gender (*P* = 0.065) or age (*P* = 0.733) between patients and controls. These demographic factors were all controlled for in the analysis, either because they differed between patients and controls or because they have previously been shown to be associated with adversity exposure and psychosis. A total of 17 patients (6% of the overall sample) were found to have an IQ of 70 or below which is considered be the cut-off for mild intellectual impairment.^39^ These individuals were sufficiently cognitively able to complete the assessments and thus were retained in the sample. Unfortunately, IQ data were not available on a large enough number of participants to be included as a confounder.

### Association between childhood adversity and psychotic disorder

Table 2 presents the prevalence of each type of childhood adversity for psychosis patients and controls along with the ORs of the associations with case status. All types of childhood adversity, except for sexual abuse, occurred more often among psychosis patients than unaffected controls. Following adjustment for demographic factors, only the associations between parental separation and psychosis remained statistically significant, with sexual abuse (*P* = 0.05) and parental loss (*P* = 0.06) approaching significance. These results confirm the previously demonstrated association between childhood adversity and psychosis.

**Table 2:** Prevalence of childhood adversity in psychosis patients and unaffected controls

Type of childhood adversity	Patients (*n*=285) *n* (%)	Controls (*n*=256) *n* (%)	Unadjusted OR	95% CI	*P*	Adjusted OR^a^	95% CI	*P*
Parental separation	158 (56.0)	90 (35.3)	**2.34**	1.65–3.31	<**0.001**	**1.96**	1.32–2.91	**0.001**
Parental loss	33 (11.7)	16 (6.3)	**1.99**	1.06–3.71	**0.031**	1.99	0.98–4.06	0.058
Physical abuse	65 (22.8)	39 (15.3)	**1.63**	1.05–2.53	**0.029**	1.47	0.89–2.43	0.127
Sexual abuse	41 (14.4)	28 (11.0)	1.36	0.81–2.27	0.245	1.81	1.00–3.30	0.050

Bold text indicates result statistically significant at *P* < 0.05.

CI, confidence interval; OR, odds ratio.

a Adjusted for gender, age at interview, ethnicity and level of education.

### Association between familial liability and psychotic disorder

Table 3 presents the prevalence of each type of familial liability for psychosis patients and unaffected controls along with the ORs of the associations with case status. All types of familial risk were significantly associated with psychosis in probands. Psychotic disorders were around four times more common in first-degree relatives of patients than controls, while more broadly defined mental illness (psychosis, depression or mania) was almost twice as common. This indicates that familial liability could be considered as a proxy genetic risk factor for psychosis, though it could also indicate the negative environmental effects of living with a first-degree relative who has a serious mental disorder. In both cases, familial liability might play a role in the previously demonstrated association between childhood adversity and psychosis.

**Table 3:** Prevalence of familial risk in psychosis patients and unaffected controls

Type of familial risk	Patients (*n*=224) *n* (%)	Controls (*n*=250) *n* (%)	Unadjusted OR	95% CI	*P*	Adjusted OR^a^	95% CI	*P*
Family mental illness	94 (42.0)	70 (28.0)	**1.86**	1.27–2.73	**0.002**	**1.76**	1.14–2.70	**0.010**
Family psychosis	38 (17.3)	12 (5.1)	**3.90**	1.98–7.68	<**0.001**	**4.11**	1.94–8.72	<**0.001**
Parental mental illness	65 (29.5)	49 (20.8)	**1.60**	1.04–2.45	**0.031**	**1.56**	0.97–2.49	<**0.001**
Parental psychosis	28 (12.8)	8 (3.4)	**4.20**	1.87–9.43	**0.001**	**4.71**	1.90–11.67	<**0.001**

Bold text indicates result statistically significant at *P* < 0.05.

Mental illness includes psychosis, depression, and mania. Family refers to first-degree relatives.

CI, confidence interval; OR, odds ratio.

a Adjusted for gender, age at interview, ethnicity and level of education.

### Proxy rGE for parental psychopathology and childhood adversity

In order to investigate the presence of a passive rGE, we tested whether parental psychopathology was also associated with childhood adversity in this sample. Therefore, the reported prevalence of parental mental illness and psychosis by exposure to childhood adversity for patients and controls is presented separately in Table 4. Parental psychopathology was not associated with greater exposure to any type of childhood adversity among patients in this sample. However, parental history of depression, mania or psychosis was more common among controls with, compared with those without, a history of parental separation and physical abuse, and these associations remained significant following adjustment for potential confounders. These results do not confirm the presence of a passive rGE, as a parental history of psychosis was associated with greater odds of psychotic disorder but not with greater exposure to childhood adversity among patients in this sample.

**Table 4:** Association between parental mental illness and childhood adversity in psychosis patients and unaffected controls

Type of parental psychopathology	Childhood adversity present, *n* (%)	Childhood adversity absent, *n* (%)	Unadjusted OR	95% CI	*P*	Adjusted OR^a^	95% CI	*P*
*Parental separation*								
Psychosis patients	*n* = 119	*n* = 98						
Parental mental illness	34 (28.6)	30 (30.6)	0.91	0.50–1.63	0.743	1.02	0.53–1.94	0.955
Parental psychosis	13 (11.1)	14 (14.3)	0.75	0.33–1.68	0.485	1.01	0.40–2.52	0.986
Unaffected controls	*n* = 82	*n* = 153						
Parental mental illness	25 (30.5)	24 (15.7)	**2.36**	1.24–4.47	**0.009**	**2.58**	1.30–5.11	**0.007**
Parental psychosis	5 (6.1)	3 (2.0)	3.25	0.76–13.94	0.113	4.36	0.78–24.43	0.094

*Parental loss*								
Psychosis patients	*n *= 26	*n *= 190						
Parental mental illness	10 (38.5)	54 (28.4)	1.57	0.67–3.68	0.296	1.91	0.77–4.73	0.162
Parental psychosis	5 (19.2)	23 (12.2)	1.71	0.59–4.97	0.326	2.23	0.71–6.96	0.167
Unaffected controls	*n* = 16	*n* = 219						
Parental mental illness	2 (12.5)	47 (21.5)	0.52	0.11–2.38	0.402	0.56	1.12–2.65	0.463
Parental psychosis	0 (0.0)	8 (3.6)	–	–	–	–	–	–

*Physical abuse*								
Psychosis patients	*n* = 47	*n* = 173						
Parental mental illness	14 (29.8)	51 (29.5)	1.01	0.50–2.05	0.967	1.09	0.52–2.31	0.816
Parental psychosis	7 (14.9)	21 (12.3)	1.25	0.50–3.15	0.636	1.42	0.53–3.83	0.487
Unaffected controls	*n* = 38	*n* = 196						
Parental mental illness	15 (39.5)	34 (17.3)	**3.11**	1.47–6.57	**0.003**	**3.74**	1.68–8.33	**0.001**
Parental psychosis	3 (7.9)	5 (2.55)	3.27	0.75–14.33	0.115	4.54	0.93–22.18	0.061

*Sexual abuse*								
Psychosis patients	*n* = 29	*n* = 191						
Parental mental illness	10 (34.5)	55 (28.8)	1.30	0.57–2.98	0.533	1.24	0.53–2.89	0.625
Parental psychosis	2 (7.0)	26 (13.8)	0.46	0.10–2.07	0.315	0.46	0.10–2.11	0.317
Unaffected controls	*n* = 22	*n* = 212						
Parental mental illness	8 (36.4)	41 (19.3)	2.38	0.94–6.06	0.068	1.99	0.71–5.60	0.192
Parental psychosis	2 (9.1)	6 (2.8)	3.43	0.65–18.14	0.146	1.60	0.16–15.57	0.685

Bold text indicates result statistically significant at *P* < 0.05.

Mental illness includes psychosis, depression and mania.

CI, confidence interval; OR, odds ratio.

a Adjusted for gender, age at interview, ethnicity and level of education; – indicates unable to calculate values due to at least one cell containing a zero value.

### Testing for confounding by parental psychopathology

Given that parental psychosis was shown to be strongly associated with psychosis case status, we investigated whether this form of familial risk could be a confounder in the original associations between childhood adversity and psychosis. As parental separation was the only form of adversity to be robustly associated with psychotic disorder we only investigated the impact on this association. The original association between parental separation and psychotic disorder (adjusted OR = 1.96, 95% CI: 1.32–2.91, *P* = 0.001) was only slightly attenuated when further adjusting for parental psychosis (adjusted OR = 1.64, 95% CI: 1.07–2.50, *P* = 0.022).

### Interaction between familial liability and childhood adversity

The associations between each combination of childhood adversity and family or parental mental illness and psychotic disorder along with the results of the interaction analyses are presented in Table 5. Associations were evident between parental separation and psychotic disorder regardless of whether or not participants had a family or parental history of mental illness. There was a trend for associations between physical abuse, sexual abuse and psychosis to be stronger among those with no familial liability for mental illness. However, there was no evidence of a positive additive interaction between these forms of childhood adversity and family history of mental illness.

**Table 5:** The synergistic effects of childhood adversity and familial liability to mental illness on the presence of psychotic disorder

	Association with psychotic disorder					
	
Combination of risk factors	Unadjusted OR	95% CI	*P*	Adjusted OR^a^	95% CI	*P*
*Parental loss (PL)*						
No PL and no family mental illness (FMI)	[reference]	–	–	[reference]	–	–
PL only (FMI absent)	1.53	0.71–3.27	0.276	0.92	0.31–2.80	0.896
FMI only (PL absent)	1.20	0.81–1.78	0.354	1.12	0.67–1.89	0.664
Both PL and FMI present	**3.82**	1.24–11.73	**0.019**	2.57	0.70–9.36	0.153
	ICR: 2.09, 95% CI –2.29 to 6.47, *P*=0.350			ICR: 1.52, 95% CI –1.90 to 4.93, *P*=0.384		
No PL and no parental mental illness (PMI)	[reference]	–	–	[reference]	–	–
PL only (PMI absent)	1.63	0.81–3.25	0.170	0.78	0.29–2.12	0.631
PMI only (PL absent)	1.14	0.73–1.76	0.566	0.89	0.49–1.61	0.693
Both PL and PMI present	**4.95**	1.07–22.88	**0.041**	4.85	0.91–25.64	0.063
	ICR: 3.18, 95% CI –4.43 to 10.80, *P*=0.412			ICR: 4.18, 95% CI –3.88 to 12.25, *P*=0.309		

*Parental separation (PS)*						
No PS and no family mental illness (FMI)	[reference]	–	–	[reference]	–	–
PS only (FMI absent)	**3.09**	2.02–4.72	<**0.001**	**4.14**	2.19–7.81	<**0.001**
FMI only (PS absent)	**1.90**	1.13–3.20	**0.015**	**2.25**	1.11–4.54	**0.024**
Both PS and FMI present	**2.33**	1.38–3.93	**0.002**	**2.20**	1.09–4.44	**0.028**
	ICR: –1.66, 95% CI –3.48 to 0.15, *P*=0.072			ICR: –3.18, 95% CI –6.33 to 0.04, *P*=0.047		
No PS and no parental mental illness (PMI)	[reference]	–	–	[reference]	–	–
PS only (PMI absent)	**2.86**	1.92–4.26	<**0.001**	**3.75**	2.09–6.75	<**0.001**
PMI only (PS absent)	**1.88**	1.03–3.41	**0.039**	**2.36**	1.04–5.36	**0.040**
Both PS and PMI present	**2.04**	1.14–3.64	**0.016**	1.62	0.75–3.51	0.224
	ICR: –1.70, 95% CI –3.53 to 0.14, *P*=0.069			ICR: –3.50, 95% CI –6.60 to 0.40, *P*=0.027		

*Physical abuse (PA)*						
No PA and no family mental illness (FMI)	[reference]	–	–	[reference]	–	–
PA only (FMI absent)	**2.53**	1.43–4.48	**0.001**	1.69	0.74–3.88	0.212
FMI only (PA absent)	**1.63**	1.07–2.48	**0.023**	1.59	0.90–2.82	0.113
Both PA and FMI present	1.18	0.61–2.30	0.622	0.80	0.34–1.91	0.617
	ICR: –1.97, 95% CI –3.74 to 0.21, *P*=0.028			ICR: –1.48, 95% CI –3.29 to 0.33, *P*=0.109		
No PA and no parental mental illness (PMI)	[reference]	–	–	[reference]	–	–
PA only (PMI absent)	**2.28**	1.34–3.86	**0.002**	1.53	0.71–3.30	0.280
PMI only (PA absent)	1.61	0.99–2.60	0.054	1.48	0.76–2.86	0.250
Both PA and PMI present	1.00	0.47–2.13	0.999	0.67	0.26–1.76	0.419
	ICR: –1.88, 95% CI –3.49 to –0.27, *P*=0.022			ICR: –1.33, 95% CI –3.00 to 0.34, *P*=0.118		

*Sexual abuse (SA)*						
No SA and no family mental illness (FMI)	[reference]	–	–	[reference]	–	–
SA only (FMI absent)	1.73	0.90–3.33	0.101	2.32	0.87–6.20	0.092
FMI only (SA absent)	1.41	0.95–2.11	0.091	1.33	0.78–2,28	0.298
Both SA and FMI present	1.19	0.54–2.66	0.663	1.33	0.46–3.81	0.596
	ICR: –0.95, 95% CI –2.49 to 0.60, *P*=0.231			ICR: –1.32, 95% CI –4.03 to 1.38, *P*=0.337		
No SA and no parental mental illness (PMI)	[reference]	–	–	[reference]	–	–
SA only (PMI absent)	1.52	0.83–2.76	0.172	2.30	0.96–5.51	0.062
PMI only (SA absent)	1.31	0.84–2.06	0.238	1.27	0.69–2.34	0.434
Both SA and PMI present	1.22	0.47–3.17	0.678	0.93	0.26–3.31	0.908
	ICR: –0.61, 95% CI –2.16 to –0.94, *P*=0.444			ICR: –1.64, 95% CI –4.09 to 0.80, *P*=0.188		

Bold text indicates result statistically significant at *P* < 0.05.

CI, confidence interval; ICR, interaction contrast ratio; OR, odds ratio.

a Adjusted for gender, age at interview, ethnicity and level of education.

Only for parental loss and familial liability was there suggestive evidence of departure from additivity (namely a stronger association with psychotic disorder for individuals with both a family psychiatric history and parental loss) but this failed to reach statistical significance.

## Discussion

The present study investigated the role of different forms of childhood adversity and familial liability to mental illness, as well as the interaction between them, in the development of psychosis. The strongest associations between childhood adversity and psychotic disorder were found for parental separation, parental loss and physical abuse, in keeping with previous findings from an overlapping geographical area.^5,40^ Moreover, within this sample, family history of mental illness was unsurprisingly a significant risk factor for psychotic disorder. Indeed, a history of psychosis in at least one parent was four times more common among participants with psychotic disorder than community controls. There was a smaller but significant association between current or past mental illness (psychosis, depression or mania) in a first-degree relative and clinical presentation of psychosis in this sample.

However, we did not find an association between parental history of psychosis and childhood adversity among the psychosis patients and, in keeping with these findings, controlling for parental history of psychosis only resulted in a small reduction in the strength of the association between parental separation and psychotic disorder. Therefore, our results could not confirm the presence of a potential passive rGE, in which parents pass on both a genetic liability to psychosis and create an abusive environment, which has been reported in a previous study.^27^ An adoption design would be required to fully exclude the possibility of a passive rGE^16^ operating in this association but suitable samples are rarely available. Unfortunately, it was not possible in the current study to explore other forms of rGE, namely evocative or active, for example, the child’s genetic propensities eliciting harsher methods of physical punishment or making them more likely to select solitary environments.^16^

Moreover, there was no evidence for additive interactions between parental separation, physical abuse or sexual abuse in childhood and family psychiatric history in relation to the presence of psychotic disorder. This could suggest that these forms of childhood adversity may be associated with psychotic disorder independently of proxy genetic risk but might also reflect a lack of statistical power in this sample. Our findings are in line with previous studies reporting that the effect of childhood trauma on later experience of psychotic symptoms was independent of proxy genetic liability to psychosis.^1,26,27^ However, our findings suggest that this may not be the case for parental loss. Overall, our results suggest that biological and environmental risk factors are both important in the aetiology of psychosis but the effects of some forms of childhood trauma might potentially act largely independently of pre-existing genetic liability to increase risk of psychosis.

### Strengths and limitations

To our knowledge, this is the first study to explore the interplay between familial liability and various forms of childhood adversity in relation to the presence of psychotic disorder. This extends a previous report from our group that focused exclusively on maternal physical abuse.^27^ The current study has several advantages, such as the inclusion of a sample of patients that had recently presented to mental health services with a psychotic disorder, thus extending previous reports that only examined psychotic symptoms or probable psychosis in the general population.^1,23–26^ Our controls screened negative for psychotic disorder and had prevalence rates of childhood adversity similar to those reported in studies of the UK general population.^41^ The proportion of patients reporting a first-degree relative with psychosis in this sample was 17.3% which is also within the range of existing studies.^20,27^ Additionally, we used a standardised measure of adverse childhood experiences^32^ and we were able to control for the potentially confounding effects of a range of demographic characteristics.

However, we had only 25% power to detect the 5% difference in proportions exposed to parental separation among individuals with a family history (*n*=162), compared with 100% power to detect the 27% difference in those without a family psychiatric history (*n*=308). Thus, we did not have enough power to test for interactions between childhood adversity and family psychiatric history in the association with psychotic disorder. Therefore, our findings should be interpreted with caution and need to be replicated in larger samples.

We also assessed childhood adversity using retrospective self-report that might have led to bias. Retrospective assessment is commonly used in studies investigating the role of childhood risk factors in psychosis as it avoids the high expense associated with following up a very large number of participants over several decades to obtain sufficient numbers with diagnosed psychosis. Although several studies have shown some bias in retrospective reports,^42^ such bias is not considered sufficiently great to invalidate retrospective case–control studies of childhood experiences.^43^ Moreover, previous studies have demonstrated that the effect of childhood adversity on psychosis remains significant regardless of the study design^44^ and histories of childhood adversity obtained from psychosis patients appear remarkably reliable over time and unaffected by current symptoms.^34^ We also attempted to enhance the validity of the self-reported experiences by utilising the CECA-Q^32^ which elicits concrete examples of adverse experiences, has a manual to score the severity of the responses in a standardised manner (http://cecainterview.com/), and uses conservative cut-offs to ensure only severe adversity is considered in analyses. All of these factors increase the likelihood of an individual accurately remembering past adverse experiences.^43^ However, if time had permitted it would have been preferable to conduct a more in-depth interview, such as the full Childhood Experience of Care and Abuse interview,^45^ with participants to obtain more detailed information about their experiences and potentially further improve accuracy of reporting.^43^

Additionally, as only trauma occurring during childhood was investigated in this study, it is possible that other environmental risk factors such as cannabis use^46^ or trauma occurring in adulthood^47^ might demonstrate stronger associations with psychotic disorder and confound this relationship. Unfortunately, there was insufficient information within the GAP study to explore the role of cannabis use or adversity in adulthood in potentially modifying the childhood adversity–psychosis association. Ideally, large samples would allow inclusion of several environmental variables in the same model, such as cannabis use and preceding or subsequent adversity, in order to address this issue more comprehensively and to obtain a greater understanding of psychosis aetiology.

Finally, we used familial psychopathology as a proxy for genetic liability which may not have captured all of the relevant genetic influences in the participants.^48^ For instance, negative family history can include undeclared or unknown positive family history of mental illness. We supplemented the interviews with information obtained from clinical records (for the cases) but there are still likely to be familial cases that were missed. It is also possible that family members have a genetic propensity to developing mental health problems but this has not (yet) been phenotypically expressed. Family psychiatric history also captures familial effects of non-genetic origin.^17^ However, the shared familial (non-genetic) component of schizophrenia risk is estimated to account for just a small proportion of the overall trait variance (4.5–11%).^10^

Unfortunately, it was not possible in the current study to adopt more sensitive measures of genetic risk.^49^ Consequently, the impact of familial liability in this sample might have been underestimated and replication using more comprehensive molecular measures of genetic risk is needed. However, very large samples are required to identify sufficient common SNPs to explain a reasonable proportion of the genetic architecture of psychotic disorders and polygenic risk scores may not get us closer to understanding the specific mechanisms involved in G × E.^22^ A recent study showed that the excess risk of offspring having schizophrenia in families affected by psychotic, bipolar affective or other psychiatric disorder is essentially unchanged when SNP-based variation is taken into account.^50^ This provides some reassurance that the data obtained in the current study on psychiatric disorder in first-degree relatives had an adequate degree of accuracy. Nonetheless, future research using larger clinical samples and exploring whether measured genes moderate the impact of childhood adversity on the onset and course of psychotic disorders would be beneficial.

### Clinical implications and further directions of research

Our results have implications for both clinical and research practices. Given that several forms of childhood adversity have been shown in the present study to be associated with psychotic disorder regardless of the presence or absence of familial liability, preventing trauma occurring or helping children to cope better in the aftermath of exposure could potentially help to prevent the onset of psychosis. Indeed, as recently shown by Kelleher *et al*,^51^ the cessation of exposure to traumatic experiences might lead to a reduction in the incidence of psychotic experiences. Therefore, interventions focused on stopping childhood adversity or dealing with its consequences might have an impact not only on preventing the onset of psychosis but also on its longer-term course. Furthermore, research has shown that if the caregiver is perceived as unavailable, unresponsive and insensitive, this could lead to the development of an insecure attachment style in the child and to the child experiencing difficulties in relating to others.^52^ Therefore, interventions focused on helping parents with psychosis and other severe mental health problems to develop better relationships with their families and/or providing family education and support could improve their children’s attachment relationships and in turn, help children develop more positive relationships with others in adulthood.^52^ Increased social networks and perceived support may reduce the likelihood of such children developing psychosis^53^ and warrants further investigation.
